# Anti-dementia drugs: a descriptive study of the prescription pattern in Italy

**DOI:** 10.1007/s10072-022-06586-8

**Published:** 2023-01-03

**Authors:** Ilaria Ippoliti, Antonio Ancidoni, Roberto Da Cas, Andrea Pierantozzi, Nicola Vanacore, Francesco Trotta

**Affiliations:** 1 National Centre for Drug Research and Evaluation, Italian National Institute of Health, Via Giano Della Bella 34, 00162 Rome, Italy; 2 National Centre for Disease Prevention and Health Promotion, Italian National Institute of Health, Via Giano Della Bella 34, 00162 Rome, Italy; 3 Department of Public Health and Infectious Diseases, Sapienza University, Piazzale Aldo Moro, 5, 00185 Rome, Italy; 4 HTA & Pharmaceutical Economy Division, Italian Medicines Agency, Via del Tritone, 181, 00187 Rome, Italy

**Keywords:** Alzheimer’s disease, Dementia, Pharmacoepidemiology, Prescription pattern, Anti-dementia drugs

## Abstract

**Introduction:**

Acetylcholinesterase inhibitors (AChEIs) and memantine are currently the only anti-dementia drugs (ADDs) approved for treating Alzheimer’s disease (AD) in Italy. This nationwide study aims to characterize dementia drug utilization in a population > 65 years, during 2018–2020.

**Methods:**

Different administrative healthcare databases were queried to collect both aggregate and individual data.

**Results:**

ADD consumption remained stable throughout the study period (~ 9 DDD/1000 inhabitants per day). AChEI consumption was over 5 DDD/1000 inhabitants per day. Memantine consumption was nearly 4 DDD/1000 inhabitants per day, representing 40% of ADD consumption. The prevalence of use of memantine represented nearly half of ADD consumption, substantially unchanged over the 3 years. Comparing the AD prevalence with the prevalence of ADDs use, the gap becomes wider as age increases. In 2019, the proportion of private purchases of ADDs was 38%, mostly represented by donepezil and rivastigmine. In 2020, memantine was the only ADD with an increase in consumption (Δ% 19–20, 1.3%).

**Discussion:**

To our knowledge, this study represents the first attempt to investigate the ADD prescription pattern in Italy with a Public Health approach. In 2019, the proportion of ADD private purchases point out several issues concerning the reimbursability of ADDs. From a regulatory perspective, ADDs can be reimbursed by the National Health System only to patients diagnosed with AD; therefore, the off-label use of ADDs in patients with mild cognitive impairment may partially explain this phenomenon. The study extends knowledge on the use of ADDs, providing comparisons with studies from other countries that investigate the prescription pattern of ADDs.

## Background

Dementia is a complex of chronic and progressive disorders that leads to deterioration of cognitive functions and concurrent onset of behavioural and psychological symptoms (BPSD); Alzheimer’s disease (AD) represents the most common form of dementia [1, 2]. In the last two decades, mild cognitive impairment (MCI) was identified and defined as a transitional state between the cognitive changes of normal ageing and very early dementia. MCI represents a risk factor for AD, and it is characterized by isolated or multiple cognitive deficits with a very slight impairment in performing activities of daily living [3, 4]. MCI has generated a great deal from both clinical and research perspectives. The prevalence of dementia in Western countries in people over the age of 65 is nearly 8% and increases to over 20% in the 80 s [5]. In Italy, recent estimates suggest that 1.1 million people have dementia and approximately 900,000 people have MCI [5, 6]. Dementia has a huge societal and economic impact on the Italian healthcare system [7]. From a public health perspective, monitoring the frequency of dementia is essential to planning and organizing health and social services and assessing the impact of potential preventive strategies.

No therapies are available yet to halt the neurodegenerative course of dementia; however, currently approved drugs only partially control cognitive functions. In Italy, acetylcholinesterase inhibitors (AChEIs) (i.e. donepezil, rivastigmine and galantamine) and memantine (NMDA receptor antagonist) are the approved drugs for treating Alzheimer’s disease (ADDs) and are reimbursed by the Italian NHS. AChEIs are approved for the treatment of mild and moderate forms of AD, while memantine is only indicated for the moderate forms [2, 8]. Moreover, there is no evidence of any differences in efficacy between AChEIs, and evidence from a systematic review shows fewer adverse events associated with donepezil compared with rivastigmine [9].

Little is known about the long-term effects of AChEIs on cognitive decline in AD. Nevertheless, a recent study on long-term effects on cognition and mortality of AChEIs in patients with dementia (AD and mixed AD) demonstrated cognitive benefits that were modest but persisted over the 5-years follow-up [10].

Several systematic reviews investigated the effects of AChEIs in MCI patients [11–13]. However, clinical benefits for these patients remain inconsistent, and current evidence does not support the use of AChEIs for cognitive improvement [11, 14]. For these reasons, either the Food and Drug Administration (FDA) or the European Medicine Agency (EMA) did not approve AD medications for patients with MCI. However, in view of the constant attention paid in recent years to the treatment of these patients, off-label use of anti-dementia drugs is considered part of the routine clinical practice. In this context, since pharmacological interventions do not halt the disease progression, integrated management for continuity of care is a crucial issue and represents a public health priority. In recent years, international attention has been turning toward therapies that modify the natural history of AD [15, 16]. Clinical trials on new drugs with a potential disease-modifying effect, especially those targeting the beta-amyloid protein (Aβ), are underway for the treatment of AD. However, only the FDA granted conditional approval for Aβ targeting antibody aducanumab, while the applicant withdrew the marketing authorization in European Union as EMA considered the antibody ineffective at treating patients with MCI/early-AD [17–19]. However, recent trials on new Aβ targeting antibodies showed that up to 80% of individuals included were MCI patients and 50–65% of enrolled participants were treated under stable doses of AD medications, mainly with AChEIs only [18].

Since new therapies could prevent or slow down the disease progression even before it becomes clinically manifest, efforts are being made to identify the target population that might benefit from such treatment, as they will most likely be used in the early stages of cognitive disorder. Therefore, the identification of patients eligible for treatment will play an essential role in access to therapy. This nationwide Italian study aims to identify the prescription pattern of drugs used in dementia and to compare our results with a recently conducted Austrian study [20]. In addition, this study will have a closer look at some aspects related to the association of memantine and AChEIs, to support any regulatory interventions. The identification and characterization of drug users for dementia treatment may be also a supporting element for the definition of the population eligible for the new anti-Aβ therapeutics that might be soon available for the treatment of patients with MCI and/or mild AD [21–23].

## Methods

### Study design

This descriptive study aims to characterize drugs utilization for dementia treatment at the national level in the Italian population aged 65 and older with an in-depth analysis of variability at the temporal level (years 2018–2020) and geographical level by macro-areas (North, Centre, South and Islands) and to define the characteristics of prevalent users of ADDs.

Information on each drug package was tracked via unique identifier codes and the 5th level Anatomical Therapeutic Chemical (ATC) classification [24]. According to this classification, we have identified donepezil (N06DA02), rivastigmine (N06DA03) galantamine (N06DA04) and memantine (N06DX01). Analyses were also conducted at the individual level with prescription patterns of users stratified by age and gender. NHS covers costs of ADDs but only for the treatment of mild and moderate forms in patients with a confirmed diagnosis of AD and with specialist’s prescriptions (geriatrician, neurologist and psychiatrist) within the Centers for Cognitive Disorders and Dementia (CCDDs), identified by individual regions. As the cost of drugs is rather low due to the expiry of patents, it cannot be excluded that many people purchase drugs at full price with a specialist’s private prescription. Therefore, we analysed the percentage, by therapeutic category and by a single drug, of the share of private purchases over the years.

### Data source

At aggregate and individual levels, we extrapolated data from the “Medical Prescriptions Database” (MPD). All prescriptions of ADDs reimbursed by the Italian NHS and dispensed by community pharmacies were identified in the “Medical Prescriptions database” which collects patient-level data and each citizen is assigned an alphanumeric code that grants anonymity and privacy.

### Data analysis

Data were analysed in terms of the amount of use (defined daily dose (DDD)) per 1000 inhabitants per day and of prevalence of use. The mean number of DDD/1000 inhabitants per day was calculated by dividing the total number of DDDs of ADDs prescribed and dispensed during the study period (between 2018 and 2020) and the total number of inhabitants with age 65 + of the Italian population (individuals registered by the Italian National Institute of Statistics in the same period). The result was then divided by 365 and reported per thousand inhabitants. For the DDD/1000 inhabitants per day, the compound annual growth rate (CAGR) was also considered. CAGR is calculated through the nth root of the overall percentage rate where *n* is the number of years of the period considered. Therefore: $CAGR=\left(\frac{xf}{xi}\right)^\frac{1}{n}$ -1.

where x_*f*_ represents the indicator calculated in the final period, *x*_*i*_ represents the indicator calculated in the initial period.

The prevalence of use was calculated, by dividing the number of individuals receiving at least one ADD during the study period and the total number of inhabitants with age 65 + of the Italian population. In the cohort of prevalent users, as primary analysis, calculation of the intensity of use of ADDs was also carried out using indicators such as DDD per user, median DDD (value dividing the ordered distribution of DDD values into 2 equal parts), prescriptions per user and percentage of users with only one prescription. The DDD per user is an indicator of the average number of days of therapy, and it was calculated by dividing the total number of ADDs’ DDDs and the number of individuals receiving at least one prescription of ADDs during the study period. The prescriptions per user are an indicator of the intensity of use of medicine, and it was calculated by dividing the total of ADD prescriptions and the number of individuals receiving at least one prescription of ADDs during the study period. As secondary analysis, the indicator DDD per users was also calculated by excluding incident users (defined as subjects that not received any prescription of ADDs in the period preceding the index date).

All the indicators considered are stratified by demographic characteristics to identify differences by age group and gender. Private purchase is obtained as a difference between what is purchased from pharmacies (sell-in), compared to what is paid by the NHS (sell-out, i.e. the Osmed flow), considering citizens as a recipient. Data of private expenditure refer to the year 2019 to avoid bias of the private purchase for the year 2020 due to the SARS-CoV-2 pandemic. Categorical variables are shown as percentages; continuous variables are presented as mean ± standard deviation (SD) for normally distributed data and median [interquartile range, IQR] for non-normal data.

## Results

ADD consumption at national level remained almost stable through the 3 years considered (2018–2020) with ~ 9 DDD/1000 inhabitants per day (9.11 in 2018, 9.16 in 2019, 8.96 in 2020, ∆% 19–20: − 2.2%; CAGR 18–20: − 0.8%) (Table 1). There was heterogeneity across the three Italian macro-areas (8.28 in North, 10.73 in Centre and 9.48 DDD/100 inhabitants per day in South; *p* < 0.001). Overall, AChEI consumption was over 5 DDD/1000 inhabitants per day (60% represented by donepezil). Consumption of memantine was nearly 4 DDD/1000 inhabitants per day, representing 40% of the overall ADD consumption with a progressive increase in these 3 years (CAGR 18–20: + 2.6%; ∆% 19–20: + 1.3%). The largest variations over the years were observed for galantamine (CAGR 18–20: − 12.8%; ∆% 19–20: − 11.8); however, consumption of galantamine was the lowest among the ADD category.

**Table 1:** Time trends and macro-areas of consumption (DDD/1000 inhabitants per day) of Alzheimer’s disease drugs, by therapeutic category and substance in the period 2018–2020 (65 + years)

ADDs	2018	2019	2020	∆ % 19–20	CAGR % 18–20
AChEIs	5.44	5.35	5.12	− 4.3	− 3.0
Rivastigmine	2.17	2.06	1.90	− 7.8	− 6.5
Galantamine	0.16	0.14	0.12	− 11.8	− 12.8
Donepezil	3.10	3.15	3.10	− 1.7	− 0.1
Other ADDs					
Memantine	3.67	3.81	3.86	1.3	2.6
Italy	**9.11**	9.16*	**8.96**	− **2.2**	− **0.8**
North	8.13	8.28	8.05	− 2.8	− 0.5
Centre	10.85	10.73	10.75	0.2	− 0.5
South and islands	9.46	9.48	9.18	− 3.2	− 1.5

^*^From sensitivity analysis including only prevalent users: 7.92 DDD/1000 inhab. per day

Bold indicates the overall data for Italy

In 2019, among AChEIs, donepezil showed the highest prevalence (0.48%), followed by rivastigmine (0.37%) and galantamine (0.02%). In 2020, the prevalence of use of memantine represented half of ADD consumption (0.65%), with no substantial differences between 2019 and 2020 (− 0.02%) (Table 2). We performed a sensitivity analysis including only prevalent users for the year 2019 (Table 1 and Table 2). The absolute difference in terms of consumption of ADDs between prevalent users vs total users (prevlents + incidents) is small (1.24 DDD/1000 inhab per day), and this difference remains also between macro-areas.

**Table 2:** Temporal and macro-area trends in the prevalence of use (%) of Alzheimer’s disease drugs, by therapeutic category and substance in the period 2018–2020 (65 + years)

ADDs	2018	2019	2020	∆ % 19–20
AChEIs				
Rivastigmine	0.38	0.37	0.33	− 0.04
Galantamine	0.03	0.02	0.02	0.00
Donepezil	0.46	0.48	0.44	− 0.03
Other ADDs				
Memantine	0.63	0.67	0.65	− 0.02
Italy	**1.32**	1.36*	**1.28**	− **0.05**
North	1.17	1.18	1.13	− 0.14
Centre	1.58	1.70	1.57	− 0.07
South and islands	1.37	1.39	1.32	− 0.05

^*^From sensitivity analysis including only prevalent users: 0.97% of prevalence of use

Bold indicates the overall data for Italy

Stratifying by age group, exposure levels and consumption increase progressively with age (in all of the three years considered), with the prevalence of use which rose in 2019 from 0.23% in the 65–69 age category to 3.14% in the age category 85–89 and then decrease over 90 years (Table 3). ADD consumption ranged from 1.52 DDD/1000 inhabitants per day in the 65–69 age category to 21.39 DDD/1000 inhabitants per day in the 85–89 age category. In the oldest age category (90 +), the prevalence of use and consumption of ADDs was significantly reduced (prevalence of use 1.85%; DDD/1000 inhabitants per day 12.03). In 2020, the number of prevalent users decreased when compared to 2018 and 2019. Reductions in the prevalence of use and consumption of ADDs occurred in the age categories 80–84 (− 0.17%, − 0.59%, respectively), 85–89 (− 0.52%, − 3.09% respectively) and 90 + (− 0.58%, − 3.62%, respectively), while no reduction were observed in the younger age categories.

**Table 3:** Prevalence of use and consumption of Alzheimer’s disease drugs by age group and macro-areas (2018–2020 period)

Age group	DDD/1000 inhab per day			Prevalence of use, %			AD prevalence, %	Prevalence of use**/AD prevalence
	Year			Year				
	2018	2019	2020	2018	2019	2020		
65–69	1.50	1.52	1.72	0.21	0.23	0.25	0.6*	0.38 (38%)
70–74	4.23	4.20	4.65	0.62	0.63	0.67	0.9	0.70 (70%)
75–79	10.32	10.17	11.31	1.49	1.50	1.60	3.5	0.43 (43%)
80–84	18.81	18.77	18.18	2.72	2.75	2.58	8	0.34 (34%)
85–89	21.07	21.39	18.30	3.04	3.14	2.62	15.8	0.20 (20%)
90 +	12.02	12.03	8.41	1.77	1.85	1.27	20.9	0.08 (8%)

^*^Prevalence of AD of 0.6% in the study by Tognoni et al. (2005) refers to the age category 64–69

^**^Year 2019

To compare AD prevalence with ADDs prevalence of use, evidence from the literature was considered [25]. In the age group 70–74, the ratio between the prevalence of use and AD prevalence observed was 0.70, representing the least difference (30%). This difference becomes larger as age increases (75–79 years, 57%; 80–84 years, 66%; 85–89 years, 80%; 90 + years, 92%).

**Fig. 1:**
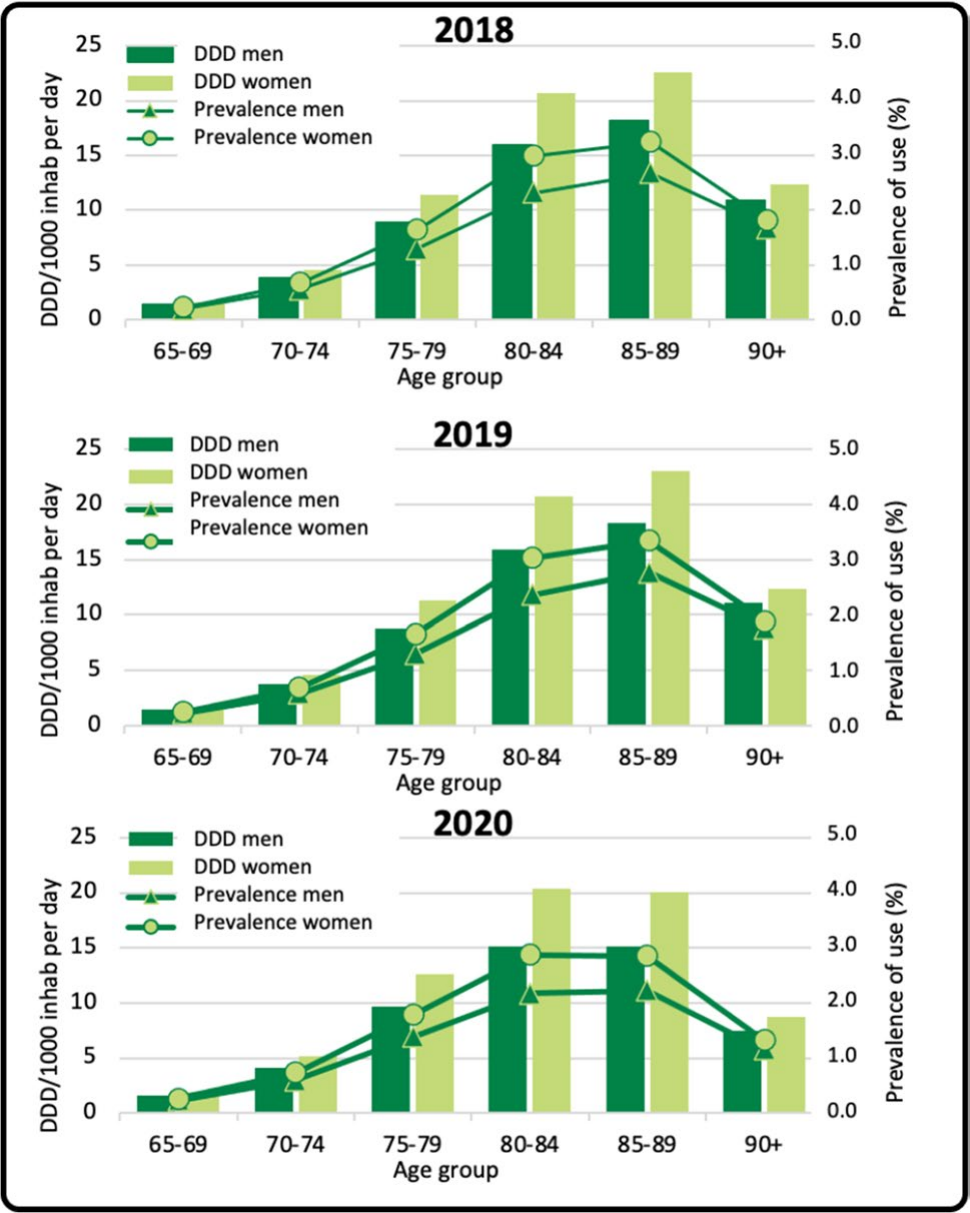
Trends in the prevalence of use and consumption of Alzheimer’s disease drugs by age group (years 2018, 2019 and 2020)

Within the 3 years considered, the prevalence of use and consumption was higher in females (+ 0.42% and 3 DDD, respectively). These differences progressively increased until the 85–89 age category (Fig. 1). The highest difference in the prevalence of use between males and females was observed in the 80–84 age category (+ 0.68% in 2018, + 0.66% in 2019 and + 0.70% in 2020). In 2020, consumption of ADDs decreased for both males and females in each age category except for 65–79 where an increment in the consumption occurred. Characterization of prevalent users was also performed and, for the year 2019, the following indicators were also considered: DDD per user, prescription per user and users with one prescription. In 2019, 185,572 ADDs users were identified with median age equal to 81 years [IQR 77–85]; most of the users were females (M/F 0.56), and the mean duration of therapy was approximately 7.5 months (Table 4). Among them, 37% received memantine, 50% at least one AChEI, while 13% a combination of two or more ADDs. In addition, a combination of AChEI and memantine or a combination of two or more AChEIs was prescribed in 11.8% and 1%, respectively. In the same year, half of the users received ADDs for less than 7.4 months (median DDD per user 224.0 [101.7–360.0]). During the same year, each user received a mean of 7.4 prescriptions, while 10.5% were the user with only one prescription. The combination of AChEIs and memantine was the highest in terms of DDD per user (464.0 [280.0–653.3]) and prescriptions per user (13.5), as a result of stable and prolonged therapies during 2019 (Table 4). It is noteworthy that, in 2020, any variation in the duration of therapy was not observed since, to guarantee the continuity of therapy during the lockdown period due to the coronavirus disease (COVID-19) pandemic, the Health Authority prolonged the validity period of ADD prescriptions. As regards the combination of AChEIs and memantine, an increase in DDD per user was observed in 2019 (median DDD per user 491.2 [IQR, 311.2–672.0]). However, in the same year, an increase in the number of prescriptions per user was observed due to a reduction in the number of prevalent users. Notable that in 2019, the number of users with one prescription decreased for all ADDs. As regards the private purchase, 38% of ADDs were not reimbursed, particularly donepezil and rivastigmine (40.9% and 27.9%, respectively) (Table 5).

**Table 4:** Prescription pattern in prevalent users (2019) of Alzheimer’s disease drugs

Macro-areas*	Prevalent users, *n* (%)	Prevalence of use^ (%)	DDD/1000 inhab per day	Median age [IQR]	Male/ female ratio	DDD per user		Prescriptions per user	Users with one prescription (%)
						Mean (SD)	Median [IQR]		
North	77,756 (41.9)	1.18	8.28	82 [78–86]	0.55	255.7 (180.6)	224.0 [112.0–364.0]	7.0	9.7
Centre	47,792 (25.8)	1.70	10.73	82 [78–86]	0.56	229.8 (181.5)	196.0 [74.7–336.0]	7.4	13.2
South and islands	60,019 (32.3)	1.39	9.48	81 [76–85]	0.55	248.5 (171.6)	224.0 [112.0–360.0]	7.8	9.4
ADDs									
Rivastigmine	39,390 (21.2)	0.29	1,58	81 [77–85]	0,57	201.1 (143.5)	174.3 [72.6–328.1]	6.6	11.3
Galantamine	2242 (1.2)	0.02	0.11	82 [78–86]	0.55	237.5 (161.8)	196.0 [112.0–364.0]	7.9	9.6
Donepezil	51,252 (27.6)	0.37	2.45	81 [77–85]	0.53	239.0 (162.5)	224.0 [74.7–373.3]	6.7	11.4
Memantine	69,163 (37.3)	0.51	2.88	82 [78–86]	0.58	207.9 (126.1)	196.0 [84.0–336.0]	6.3	13.0
AChEI + memantine	21,805 (11.8)	0.16	2.06	81 [76–85]	0.53	471.4 (238.2)	464.0 [280.0–653.3]	13.5	**0.0
Donepezil + rivastigmine	1556 (0.8)	0.01	0.08	79 [75–83]	0.54	244.3 (168.2)	207.4 [122.4–329.3]	8.5	0.0
Galantamine + rivastigmine	91 (0.05)	< 0.005	< 0.005	80 [76.5–84]	0.49	224.0 (140.9)	182.5 [115.7–340.0]	9.0	0.0
Donepezil + galantamine	71 (0.04)	< 0.005	< 0.005	79 [75–84]	0.61	281.5 (198.4)	205.3 [130.7–420.0]	9.5	0.0
Donepezil + galantamine + rivastigmine	7 (< 0.005)	< 0.005	< 0.005	84 [82–87.5]	0.75	233.5 (86.2)	208.5 [168.5–331.3]	10.1	0.0
Total ADDs	**185,572 (100)**	**1.36**	**9.16**	**81 [77–85]**	**0.56**	**246.7 (178.3)**	**224.0** **[101.7–360.0]**	**7.4**	**10.5**

^*^In five patients, no macro-area information was available

^**^Combination of two or more ADDs do not allow to identify users with one prescription since the therapy requires a single prescription for each active principle

^ANOVA test: *p* < 0.001 North vs Centre and North vs South

Bold indicates the overall data for ADDs

**Table 5:** Alzheimer’s disease drug consumption reimbursed by NHS (territorial and public pharmacies) and percentage of private purchase in the general population (2019)

ADDs	Reimbursed by NHS	Private purchase	Percentage of private purchase**
	DDD/1000 inhab per day	DDD/1000 inhab per day	
AChEIs	0.40	0.24	37.5
Rivastigmine	0.11	0.04	27.9
Galantamine	0.01	< 0.005	25.0
Donepezil	0.28	0.19	40.9
Other ADDs			
Memantine	0.28	0.17	13.1
Total ADDs	**0.68**	**0.41**	**37.9**

^**^Private purchase is obtained as a difference between what is purchased from pharmacies (sell-in, pharmaceutical companies feed this database), compared to what is paid by the NHS (sell-out, i.e. the Osmed flow), considering citizens as a recipient. It should be noted that when analysing the consumption related to a wide time span, any misalignment between sell-in and sell-out is minimized, consequent to the re-composition of the warehouse stocks of the pharmacy, which on the contrary could affect significantly on the single month

Bold indicates the overall data for ADDs

## Discussion

To our knowledge, this is the first study aiming to investigate the prescription pattern of ADDs throughout the Italian territory. Several findings from this study require further comments. First, this study observed that ADD consumption and prevalence of use remained almost stable throughout the period 2018–2020 with a substantial heterogeneity across the Italian macro-areas. Our findings are consistent with previous studies [20, 26, 27]. Memantine consumption represented nearly 40% of the overall ADD consumption with a progressive increase during the study period. In 2020, the number of users with one prescription decreased for all ADDs. This could be the result of the decrease of users, who started therapy (incident users) in the last quarter of the year (last observation on December 31st, 2020), caused by limited access to CCDDs due to COVID-19. This result could be also related to an increase in private purchase, users’ hospitalization, or users’ death during the pandemic [28, 29]. These factors may have played a role in both the reduction of prescriptions and the total number of users. In 2020, consumption of ADDs decreased in older age categories, while for 65–79, an increment of ADD consumption was observed. This is possibly related to an increase in the consumption of memantine [30], the only one ADD with an increase in consumption in 2020. An explanation of the increment of ADD consumption in younger age categories could be that these individuals are involved in psycho-educational activities that were interrupted during 2020. Hence, ADD prescribers, to prevent faster cognitive decline and to manage BPSD, may have increased the consumption of memantine, the only ADD that showed some benefits against BPSD [30, 31]. In individuals older than 75 years, a progressively wider gap is observed between the prevalence of ADD use and AD prevalence. Several factors such as comorbidities and then polytherapy, with an increased risk of interactions, adverse events and therapy failures may explain this phenomenon. Additionally, the ineffectiveness of ADDs with the disease progression may induce specialists to interrupt the prescription of these drugs. In addition, elderly patients may find more challenging to have access to CCDDs, thereby leading to a reduction in ADD consumption. Regarding the use of AChEIs and memantine together, the median DDD per user ranged between 464 and 491 DDD during the 3 years. However, we could not discern between switches and concomitant treatments; therefore, we can only speculate on the concomitant treatment of AChEIs and memantine. Despite AChEIs and memantine have shown some benefits when prescribed together [32], prescribing information currently does not indicate any combination therapy between AChEIs and memantine since evidence from RCTs does not highlight any long-term clinical benefits deriving from this combination [31]. However, at national level, there is no indication against the reimbursement of AChEIs and memantine in combination [8]. In addition, recent guideline recommendations consider the possibility to use memantine and AChEIs in combination in both moderate and severe forms of AD. Moreover, ADDs as off-label medications are recommended even for other types of dementia such as dementia with Lewy bodies and mixed dementia [33]. For this reason, at the national level, it cannot be ruled out that, to allow reimbursement of ADDs, patients with dementia from other aetiology might receive an AD diagnosis. In 2019, the private purchase of ADDs (the proportion of ADDs not reimbursed by the NHS) was 38%. This data points out several issues concerning the reimbursability of ADDs on the Italian territory. The off-label use of ADDs in patients with MCI may partially explain this proportion of private purchases. From a regulatory perspective, ADDs can be reimbursed by the NHS only to patients diagnosed with AD and with a prescription from CCDDs [8].

Recent data retrieved by a national survey on 577 CCDDs underlined that there is an evident inhomogeneity in the territorial distribution of CCDDs in Italy [34]. This can delay or prevent timely access to care services. This lack of homogeneity even in terms of different opening hours/days, and different staff compositions could play a key role in determining profound differences in the provision of dementia care services [34]. These factors could concur to increase the waiting time from referral to the first visit or to receive a new or renewal of a pharmaceutical prescription. Hence, AD patients may resort to private purchase to maintain their continuity of treatment, consequently reducing the proportion of ADDs reimbursed by the NHS.

We believe that this study has some strengths. First, our study represents an opportunity to define the prescription pattern of ADDs throughout the Italian territory. In particular, through administrative databases, we collected all prescriptions of ADDs among all Italian citizens, analysing data from both aggregate and individual perspectives. A comparison of two European countries, demographically similar, shows that the prevalence of ADDs use, across different years, was similar, nearly 1% in Austria in 2014–2015 [20] and 1.3% in Italy in 2018–2019. Our study showed that, in each age category, female patients were more frequently treated than males, as expected from literature findings regarding AD prevalence [20, 26, 27].

In addition, to our knowledge, this is the first study that compared the prescription pattern of ADDs with the prevalence of AD. We believe that this comparison shed light on important discrepancies between the prevalence of AD and ADD consumption.

However, some limitations need to be explored. First of all, in our study, we did not identify patients that received the first pharmacological prescription of ADDs as any in-depth analysis regarding the incidence of use and consumption was not part of our primary objectives. As a consequence, we were not able to characterize the socio-demographic characteristics of incident users. Moreover, we had no clinical data available since no record linkage with other healthcare databases was possible to perform. Hence, we could not perform any assessment between ADD prescription and disease severity. However, through prevalent data, we observed a reduction in the prevalence of use and consumption of ADDs which becomes more pronounced with increasing age. This could be probably ascribed to the suspension of any ADD prescription, as current drugs are likely to be considered clinically ineffective in advanced disease stages and less safe when administered with concomitant therapies. As a further limitation, we only included patients older than 65 in our study. However this cut-off is commonly used to select people with dementia. Moreover, from a further analysis in people with early onset dementia (age range 50–64), we did not observe significant values in terms of prevalence of use and DDD/1000 inhabitants per day.

Although our study was the first one to compare AD prevalence with the prevalence of use of ADDs [25], some limitations of our approach need to be addressed: first, the study by Tognoni et al. [25] is an outdated article; thus, it suffers from several biases mainly resulting from new diagnostic criteria; second, this was a door-to-door study of a district in a central region of Italy; therefore, it is not representative of the Italian population. However, to our knowledge the study by Tognoni et al. [25] was the only one study to report the prevalence of AD dementia. A recent real-life data study on the use of AchEIs and memantine in Hungary confirmed our findings showing that the prevalence of use of ADDs is not a proxy for the AD dementia prevalence [35]. An in-depth analysis of the literature on the prevalence of AD in Italy did not allow us to identify nationwide epidemiological studies providing more accurate estimates of the disease prevalence in our country [36].

## Conclusions

This is the first study investigating the prescription pattern of drugs approved for the AD condition using administrative databases throughout the Italian territory with a public health approach. The study extends knowledge on the use of ADDs, providing useful comparisons with studies that investigate the prescription pattern of these drugs in other countries. The utilization of prescription databases will allow us to investigate the prescription pattern of ADDs with a deeper analysis of medication discontinuation, switches and concomitant therapies (e.g. antipsychotics). A record linkage of prescription databases at the regional level with hospital discharge forms databases will allow us to provide an in-depth analysis of patients with MCI. Hence, the identification of a cohort of MCI patients will be propaedeutic to investigate which is the prescription pattern of ADDs in this condition. Moreover, further studies investigating the safety and efficacy of ADDs in a real-world setting need to be conducted to better describe the clinical features of the current users. We consider as a crucial aspect characterizing the real-world population that receives ADDs to support Regulatory Authorities to identify eligible patients for new disease-modifying therapies. These topics will be further evaluated in our forthcoming studies.

## Data Availability

The data that support the findings of this study are available from the corresponding author, AA, upon reasonable request.

## References

[1] Global action plan on the public health response to dementia 2017–2025. Geneva: World Health Organization; 2017. Licence: CC BY-NC-SA 3.0 IGO

[2] The Medicines Utilization Monitoring Centre. National Report on Medicines use in Italy. Year 2020. Rome: Italian Medicines Agency, 2021

[3] Petersen RC, Smith GE, Waring SC, Ivnik RJ, Tangalos EG, Kokmen E (1999). Mild cognitive impairment: clinical characterization and outcome. Arch Neurol.

[4] Petersen RC, Negash S (2008). Mild cognitive impairment: an overview. CNS Spectr.

[5] Bacigalupo I, Mayer F, Lacorte E, Di Pucchio A, Marzolini F, Canevelli M, Di Fiandra T, Vanacore N (2018). A systematic review and meta-analysis on the prevalence of dementia in Europe: estimates from the highest-quality studies adopting the DSM IV diagnostic criteria. J Alzheimers Dis.

[6] Sachdev PS, Lipnicki DM, Kochan NA, Crawford JD, Thalamuthu A, Andrews G, Brayne C, Matthews FE, Stephan BC, Lipton RB, Katz MJ, Ritchie K, Carrière I, Ancelin ML, Lam LC, Wong CH, Fung AW, Guaita A, Vaccaro R, Davin A, … Cohort Studies of Memory in an International Consortium (COSMIC) (2015) The prevalence of mild cognitive impairment in diverse geographical and ethnocultural regions: the COSMIC Collaboration. PloS one 10(11):e0142388. 10.1371/journal.pone.0142388 10.1371/journal.pone.0142388 PMC4634954 26539987

[7] http://www.alzheimer-aima.it/img/iniziative/Aima-Censis-24-febbraio_Sintesi-dei-risultati.pdf. Published February 2016. Accessed February 13, 2022

[8] https://www.aifa.gov.it/nota-85. Agenzia Italiana del farmaco. Nota 85 erogazione a carico del servizio sanitario nazionale dei farmaci rivastigmina, memantina, galantamina, donepezil. Accessed May 23, 2022

[9] Birks JS. Cholinesterase inhibitors for Alzheimer’s disease. Cochrane Database of Systematic Reviews 2006, Issue 1. Art. No.: CD005593. 10.1002/14651858.CD005593. Accessed 14 February 2022 10.1002/14651858.CD005593 PMC9006343 16437532

[10] Xu H, Garcia-Ptacek S, Jönsson L, Wimo A, Nordström P, Eriksdotter M (2021). Long-term effects of cholinesterase inhibitors on cognitive decline and mortality. Neurology.

[11] Matsunaga S, Fujishiro H, Takechi H (2019). Efficacy and safety of cholinesterase inhibitors for mild cognitive impairment: a systematic review and meta-analysis. J Alzheimers Dis.

[12] Raschetti R, Albanese E, Vanacore N, Maggini M (2007). Cholinesterase inhibitors in mild cognitive impairment: a systematic review of randomised trials. PLoS Med.

[13] Fitzpatrick-Lewis D, Warren R, Ali MU, Sherifali D, Raina P (2015). Treatment for mild cognitive impairment: a systematic review and meta-analysis. CMAJ Open.

[14] O'Brien JT, Holmes C, Jones M, Jones R, Livingston G, McKeith I, Mittler P, Passmore P, Ritchie C, Robinson L, Sampson EL, Taylor JP, Thomas A, Burns A (2017). Clinical practice with anti-dementia drugs: a revised (third) consensus statement from the British Association for Psychopharmacology. J Psychopharmacol (Oxford, England).

[15] Schneider L (2020). A resurrection of aducanumab for Alzheimer’s disease. Lancet Neurol.

[16] Musiek ES, Morris JC (2021). Possible consequences of the approval of a disease-modifying therapy for Alzheimer disease. JAMA Neurol.

[17] https://www.fda.gov/drugs/postmarket-drug-safety-information-patients-and-providers/aducanumab-marketed-aduhelm-information. Published June 2021. Accessed February 14, 2022.

[18] https://www.fda.gov/media/143502/download. Published November 6, 2020. Accessed February 14, 2022.

[19] https://www.ema.europa.eu/en/medicines/human/withdrawn-applications/aduhelm. Published April, 22, 2022. Accessed May 23, 2022.

[20] Wurm R, Stamm T, Reichardt B, Schwarz F, Parvizi T, Silvaieh S, König T, Cetin H, Stögmann E (2020). Prescription patterns of antidementives in a high income country: a pharmacoepidemiologic study. Alzheimers Dementia (NY).

[21] Panza F, Lozupone M, Logroscino G, Imbimbo BP (2019). A critical appraisal of amyloid-β-targeting therapies for Alzheimer disease. Nat Rev Neurol.

[22] Avgerinos KI, Ferrucci L, Kapogiannis D (2021). Effects of monoclonal antibodies against amyloid-β on clinical and biomarker outcomes and adverse event risks: a systematic review and meta-analysis of phase III RCTs in Alzheimer's disease. Ageing Res Rev.

[23] Lacorte E, Ancidoni A, Zaccaria V, Remoli G, Tariciotti L, Bellomo G, Sciancalepore F, Corbo M, Lombardo FL, Bacigalupo I, Canevelli M, Piscopo P, Vanacore N (2022). Safety and efficacy of monoclonal antibodies for Alzheimer’s disease: a systematic review and meta-analysis of published and unpublished clinical trials. J Alzheimers Dis.

[24] https://www.whocc.no/atc_ddd_index/2021. World Health Organization. Anatomical Therapeutic Chemical (ATC) Classification. Accessed February, 16, 2022

[25] Tognoni G, Ceravolo R, Nucciarone B, Bianchi F, Dell'Agnello G, Ghicopulos I, Siciliano G, Murri L (2005). From mild cognitive impairment to dementia: a prevalence study in a district of Tuscany, Italy. Acta Neurol Scand.

[26] Johnell K, Religa D, Eriksdotter M (2013). Differences in drug therapy between dementia disorders in the Swedish dementia registry: a nationwide study of over 7,000 patients. Dement Geriatr Cogn Disord.

[27] Bohlken J, Jacob L, van den Bussche H, Kostev K (2018). The influence of polypharmacy on the initiation of anti-dementia therapy in Germany. J Alzheimers Dis.

[28] Bianchetti A, Rozzini R, Guerini F, Boffelli S, Ranieri P, Minelli G, Bianchetti L, Trabucchi M (2020). Clinical presentation of COVID-19 in dementia patients. J Nutr Health Aging.

[29] Hwang JM, Kim JH, Park JS, Chang MC, Park D (2020). Neurological diseases as mortality predictive factors for patients with COVID-19: a retrospective cohort study. Neurol Sci.

[30] Maidment ID, Fox CG, Boustani M, Rodriguez J, Brown RC, Katona CL (2008). Efficacy of memantine on behavioral and psychological symptoms related to dementia: a systematic meta-analysis. Ann Pharmacother.

[31] McShane R, Westby MJ, Roberts E, Minakaran N, Schneider L, Farrimond LE (2019). Memantine for dementia. Cochrane Database Syst Rev.

[32] Rountree SD, Atri A, Lopez OL, Doody RS (2013). Effectiveness of antidementia drugs in delaying Alzheimer’s disease progression. Alzheimers Dement.

[33] Dementia: assessment, management and support for people living with dementia and their carers NICE guideline [NG97] Published: 20 June 2018. https://www.nice.org.uk/guidance/ng97. Accessed 26 June 2022 30011160

[34] Canevelli M, Di Pucchio A, Marzolini F, Mayer F, Massari M, Salvi E, Palazzesi I, Lacorte E, Bacigalupo I, Di Fiandra T, Vanacore N (2021). A national survey of centers for cognitive disorders and dementias in Italy. J Alzheimers Dis.

[35] Balázs N, Bereczki D, Ajtay A, Oberfrank F, Kovács T (2022). Cholinesterase inhibitors for the treatment of dementia: real-life data in Hungary. Geroscience.

[36] Bruti G, Cavallucci E, Mancini M, Bitossi A, Baldereschi M, Sorbi S (2016). A systematic review of the quality of studies on dementia prevalence in Italy. BMC Health Serv Res.

